# Supplementary figures and images for: Direct interaction with the BRD4 carboxyl-terminal motif (CTM) and TopBP1 is required for human papillomavirus 16 E2 association with mitotic chromatin and plasmid segregation function
Source: J Virol. 2023 Sep 15;97(10):e00782-23. doi: 10.1128/jvi.00782-23 (PMC10617519; doi:10.1128/jvi.00782-23)

**A**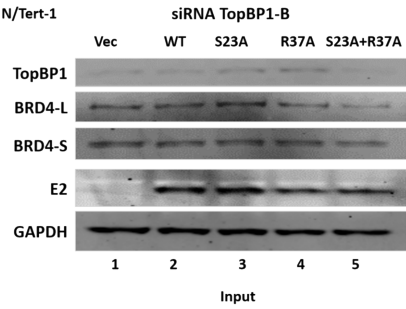**B**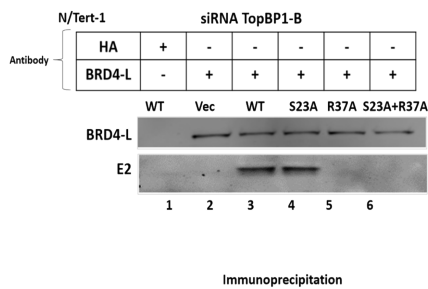**C**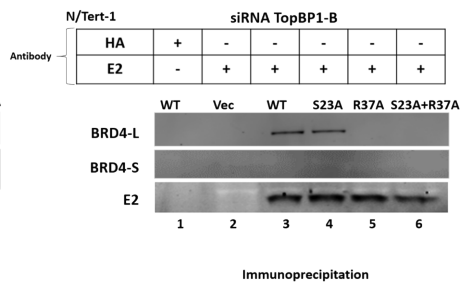**D**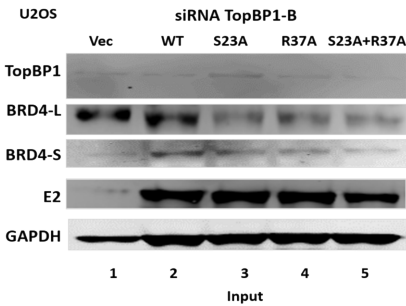**E**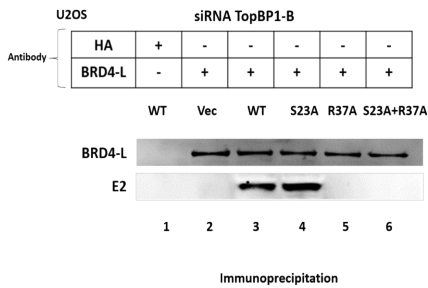**F**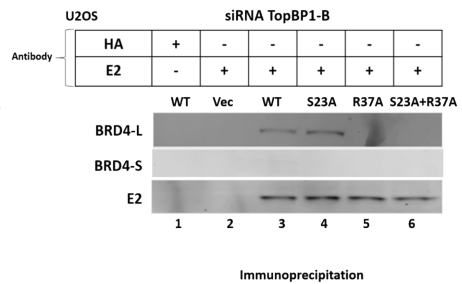

Supplement: Figure S1 — This is a repeat of the TopBP1 siRNA experiment. [file jvi.00782-23-s0001.pdf]
